# ZnO@ activated carbon derived from wood sawdust as adsorbent for removal of methyl red and methyl orange from aqueous solutions

**DOI:** 10.1038/s41598-024-55158-7

**Published:** 2024-03-05

**Authors:** Nessma S. M. Sayed, Abdelaal S. A. Ahmed, Mohamed H. Abdallah, Gamal A. Gouda

**Affiliations:** Chemistry Department, Faculty of Science, Al-Azhar University, Asyût, 71524 Egypt

**Keywords:** Environmental sciences, Materials science, Environmental chemistry, Materials chemistry, Surface chemistry

## Abstract

Activated carbon (AC) and ZnO@AC composite derived from wood sawdust were prepared to be utilized as adsorbents for methyl red (MR) and methyl orange (MO) anionic dyes from the aqueous solutions. The maximum adsorption capacity of the AC and ZnO@AC composite toward both dyes was achieved in the strong acidic medium (pH = 3), and under stirring for 60 min. The kinetic studies revealed that the adsorption of MR and MO dyes onto the AC and ZnO@AC composite fitted well with the pseudo-second-order model. Furthermore, the intraparticle diffusion and Elovich kinetic models confirmed the adsorption is controlled by external surfaces, and the adsorption is chemisorption process. The isotherm results indicated that the MR and MO dye adsorption occurred via monolayer adsorption, and the estimated maximum adsorption capacities of both dyes onto the ZnO@AC composite were higher than those achieved by AC. Thermodynamic analysis suggested that the adsorption is endothermic and spontaneous. The mechanism for MR, and MO dyes adsorption onto the AC and ZnO@AC composite is proposed to be controlled by electrostatic bonding, π–π interactions, and ion exchange, while H-bonding and n–π interactions were minor contributors. This study reveals the potential use of carbon-based adsorbents derived from wood sawdust for the removal of anionic dyes from wastewater.

## Introduction

The growing global population combined with the scarcity of freshwater supplies is a serious environmental issue. Due to increasing water usage, massive quantities of organic and inorganic contaminants, including pesticides, heavy metals, detergents, and dyes, have drastically grown in all types of water resources. For many years, a variety of industries, including paper, food, medicine, tannery, textile dying, and pigmentation, have created large amounts of colored effluents. More than 1.6 million tons of dyes are generated annually, with 10–15% ending up in wastewater disposal^1^. The dyeing effluents often contain high chemical and biochemical oxygen demand (COD and BOD) values and high color strength^2^. Discharging water without being properly treated is considered a big challenge due to its toxicity and carcinogenicity. Additionally, most aromatic dyes are non-biodegradable and have short-and long-term effects on aquatic life and humans. Most dyes are poisonous, non-biodegradable, and stable under chemical and heat conditions. The presence of dyes in water prevents sunlight from penetrating, which is a serious problem that leads to the eutrophication of water bodies and the demise of plants and animals. Thus, developing effective methods to remove artificial colors from water is highly desired^3^. Various techniques, including sono-photocatalytic degradation^4,5^, biological treatment^6^, and so on can be used for the elimination of dyes from wastewater. These methods do, however, have a number of shortcomings, including poor recovery, restricted selectivity, and high operating and maintenance costs. Due to its high efficiency, ease of use and design, low cost, lack of by-products, and quick processing time, adsorption is a promising method for removing dye from aqueous solutions^3,7^. Till now, a wide range of materials, including activated carbons (Acs)^8^, magnetic based nanomaterials^9^, and polymers^10^, have been effectively used as adsorbents to remove organic dyes. Among these, activated carbon (AC) has been demonstrated by the US-Environmental Protection Agency (EPA) as one of the most effective environmental management technologies due to its porous structure and substantial surface area^11^. In wastewater treatment, AC is thought to be an efficient adsorbent for a wide range of organic and inorganic pollutants. However, commercial AC is expensive, thus, there has been a lot of interest lately in preparing AC from low-cost naturally occurring sources. Agricultural waste is one of the possible inexpensive starting materials for the production of AC^8^. Many studies have utilized sawdust as a precursor to prepare AC for dye removal from aqueous solutions, as reported recently by Gupta et al.^12^ and Hanafiah et al.^13^ Unfortunately, the low surface area of these sawdust-derived ACs results in a low adsorption capability and a time-consuming adsorption procedure, severely limiting their practical application. As a result, the surface area and adsorption of sawdust derived ACs need to be increased. Impregnation of metal oxides is an effective strategy for improving the overall adsorption properties of AC^8^. This is due to the enormous surface area and strong physical and chemical influences on the adsorption of dye effluents. Metal oxides have been extensively used as promising adsorbents to eliminate synthetic dyes from wastewater^14^. Zinc oxide nanoparticles (ZnO NPs) widely utilized in solar cells^15^, energy storage^16^, and gas sensors^17^. Also, ZnO NPs displayed great attention in wastewater treatment, due to their high adsorptive capacity toward various pollutants^18^. Many studies have reported that ZnO NPs have displayed a promising adsorptive toward the organic dyes. For example, Zafar et al., reported that the adsorption of methyl orange (MO) and amaranth (AM) onto ZnO NPs was fitted with the Langmuir adsorption model and the pseudo-second-order kinetic model^19^. The maximum adsorption capacities have been achieved at a pH of 6. Furthermore, Zhang et al.^20^, reported that ZnO NPs showed promising adsorption ability toward cationic dyes (malachite green (MG)) and anionic dyes (acid fuchsin (AF), Congo red (CR)). The maximum adsorption capacity was 2963, 3307, and 1554 mg/g for MG, AF, and CR, respectively. This pushes us to prepare ZnO NPs dispersed into AC-derived from wood sawdust (ZnO@AC composite). The prepared AC and ZnO@AC composite were characterized and then applied as adsorbents toward MR and MO as anionic dyes. All factors affecting the adsorption performance, such as solution pH, contact time, amount of adsorbent (dose), and initial dye concentrations, were systematically investigated, and discussed. In addition, kinetics, isotherms, and thermodynamic characteristics were studied. The findings in our study demonstrate that the prepared AC and ZnO@AC composite exhibits good adsorption capacity toward MR and MO dyes, and the adsorption process is chemisorption. Furthermore, the prepared materials displayed good reusability, suggests their potential to be utilized as effective adsorbents in wastewater treatment.

## Experimental section

### Materials

All chemicals used in this study were of analytical grade. Phosphoric acid (H_3_PO_4_, ≥ 85 wt.% in H_2_O), sodium hydroxide (NaOH, ≥ 98%, pellets anhydrous), hydrocholoric acid (HCl, 37%), zinc acetate dihydrate (Zn(CH_3_COO)_2_·2H_2_O, ≥ 98%), ammonia solution (NH_3_.OH, anhydrous ≥ 99.98%), ethanol, absolute alcohol (C_2_H_5_.OH, ≥ 95%), methyl red (C_15_H_15_N_3_O_2_), and methyl orange (C_14_H_14_N_3_O_3_SNa) with the highest purity from Merck, Darmstadt, Germany. All used reagents were of analytical purity and used as received.

### ***Preparation of adsorbent materials***

**Figure 1:**
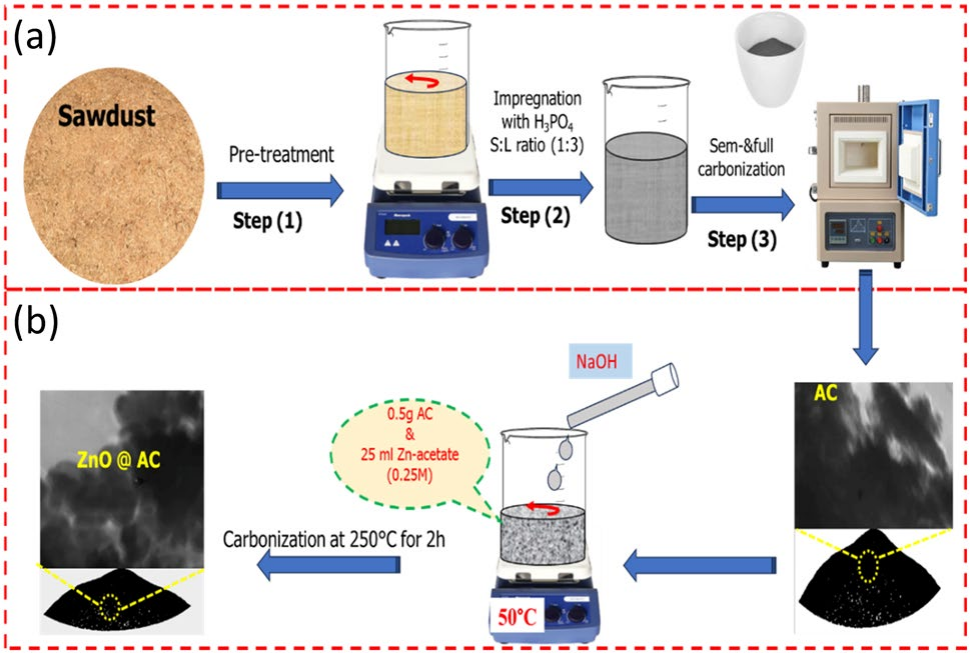
Scheme for the preparation of the (**a**) AC and (**b**) ZnO@AC composite derived from wood sawdust.

Here, the AC and ZnO@AC composite derived from wood sawdust were prepared by carbonization method. The sawdust was obtained from a wood carpentry workshop in Assiut Governorate, Egypt. The details for preparation of the AC and ZnO@AC composite are described in Fig. 1. For the preparing AC derived from sawdust as described in Fig. 1a, the sawdust was first washed thoroughly with hot tap water to remove any dust and impurities and once with distillated water, followed by drying at 105 °C for 12 h. Then, 2 g of dried powder was soaked in concentrated H_3_PO_4_ in a 1:3 (S:L) ratio and kept without starrier at room temperature for 12 h. The sample was subjected to semi-carbonization for 5 h at 250 °C, followed by full carbonization at 600 °C for 3 h. Then, the obtained powder was impregnated into a 0.1 M NaOH solution to remove the excess H_3_PO_4_, followed by washing with distilled water until the pH of the filtrates reached a constant value. Finally, the sample was dried at 105 °C and sieved, and the obtained AC was stored in tightly closed bottles.

Figure 1b represents the procedure for preparing ZnO@AC composite derived from wood sawdust. Simply 0.5 g of the AC was impregnated in 25 mL aqueous solution of zinc-acetate (0.25 mol/L) and kept under magnetic stirring at 50 °C for 1 h. Then, an aqueous solution of NaOH (0.5 mol/L) was dropped onto the above mixture, and the mixture was kept under a magnetic stirrer for another 2 h. Then, the mixture was subjected to centrifugation, and the solid particles were thoroughly washed with distilled water until the pH of the filtrate reached a constant value. Finally, the sample was dried at 100 °C overnight and subjected to carbonization at 250 °C for 2 h. The obtained ZnO@AC composite derived from wood sawdust was stored in tightly closed bottles.

### Characterization

The AC and ZnO@AC composite derived from wood sawdust were characterized by various techniques. Fourier transform infrared spectroscopy (FTIR) was carried out to identify the functional groups of the synthesized materials with and without adsorbed dyes. The FTIR spectra was collected in the 4000–400 cm^−1^ regions at a resolution of 2 cm^−1^ on a Nicolet spectrophotometer (model 6700) by the KBr pellet technique. The samples were prepared by mixing quite a small amount of the powder with KBr in 1:100 (w/w) followed by compressing into thin pellets for FTIR analysis. The crystallinity of the prepared materials was investigated by X-ray diffraction (XRD). The XRD patterns were obtained by a Bruker D8 advanced X-ray diffractometer with a monochromatized Cu-Kα radiation source operated at 40 kV. The surface area was investigated by nitrogen adsorption–desorption on Micromeritics ASAP 2020 HD88 system. The surface area and the pore size distribution were determined by Brunauer–Emmett–Teller (BET) and Barrett-Joyner-Halenda (BJH) models, respectively.

The pH values of the prepared materials at the point of zero charge (pH_PZC_) were determined using the solid addition method, which is analogous to the drift method^21^. Briefly, the pH_pzc_ experiments were performed in the absence of dye in 50 mL flasks wrapped with aluminum foil that contained 20 mL of 0.1 M NaCl solutions. The initial pH (${\text{pH}}_{{\text{i}}}$) values were adjusted from 2 to 12 by 0.1 M HCl and 0.1 M NaOH solutions. To each solution, 0.05 g of absorbent (separately, the AC and ZnO@AC composite) and kept under shaking at room temperature (20 ± 2 °C) for 24 h. After that, the final pH values (${\text{pH}}_{{\text{f}}}$) of the supernatant liquid were determined. By plotting the ${\text{pH}}_{{{\text{inital}}}}$ versus the ${\text{pH}}_{{{\text{final}}}}$, the pH of the point of zero charge (pH_PZC_) was determined^22^.

The surface morphologies were examined by a field emission scanning electron microscope (SEM) JSM 7100F FESEM (Zeiss Ultra Plus) supplied with an energy dispersive spectrum (EDS) analyzer operated at 20 kV. Transmission electron microscopy (TEM) images were obtained by a JEM-2100F field emission microscope (JEOL Ltd., Japan) with an accelerating voltage of 200 kV.

### ***Adsorption study***

The adsorption processes of MR and MO dyes were performed in a 50 mL dark bottle under a magnetic stirrer. The effects of solution pH, contact time, dye concentration, adsorbent dosage, and temperature on the removal percentage of dyes by the AC and ZnO@AC composite were investigated by batch adsorption technique. In each experiment, 0.03 g of adsorbent material was mixed with 25 mL of dye solution at an initial concentration of 50 mg/L. The pH of dye solutions was adjusted by 0.1 M HCl and 0.1 M NaOH aqueous solutions. The remaining concentration of dyes was measured using a UV–visible spectrophotometer at wavelengths of 520 nm for MR and at 464 nm for MO. Similar experimental procedure were also used to investigate the influence of the initial dye concentration (10 mg/L–200 mg/L), contact time (2 min–120 min), temperature (30 °C–80 °C), solution pH (3–11), and adsorbent dosage (0.01 g–0.1 g) on the adsorption process.The removal percentage (R%) and the adsorption capacity (q_e_; mg/g) of dye were determined by Eqs. (1) and (2), respectively

1 $${\text{R }}\left( {\text{\% }} \right) = \frac{{{\text{C}}_{0} - {\text{C}}_{{\text{e}}} }}{{{\text{C}}_{0} }} \times 100$$

2 $${\text{q}}_{{\text{e}}} = \frac{{({\text{C}}_{0} - {\text{C}}_{{\text{e}}} )}}{{\text{M}}}{\text{V}}$$

where C_0_ and C_e_ are the initial and final dye concentrations (mg/L), respectively. V is the volume (L), and M is the mass of the adsorbent (g).

## Results and discussion

### Characterization of AC and ZnO@AC composite

The XRD patterns of the AC and ZnO@AC composite are presented in Fig. 1a. The pattern of AC displayed two broad characteristic peaks located at 2θ = 25.5° and 43.5°, that assigned to the (002) and (100) planes of graphite and the long-range disordered structure, respectively^23^. In addition, the absence of other peaks revealed the full carbonization of sawdust under preparing conditions. The patterns of the ZnO@AC composite are properly indexed to hexagonal wurtzite ZnO (JCPDS 36-1451) and matched well with the previously reported literature^15,24^. Furthermore, the analysis showed no extra peaks, which is due to the purity of the prepared ZnO@AC composite. The average crystallite sizes of the AC and ZnO@AC composite are determined by the Debye–Scherrer equation (Eq. 3)

3 $$D_{p} = \frac{k \times \lambda }{{\beta \times Cos\theta }}$$

where D_*p*_ is the average crystallite (grain) size (nm), K is the Scherrer constant (0.89), λ is the X-ray wavelength (1.5418 Å for Cu Kα), β is the line broadening full width at half the maximum intensity of the peak (FWHM), and θ is Bragg’s diffraction.

The estimated crystallite (grain) sizes of the AC and ZnO@AC composite are 0.67 and 14.93 nm, respectively.

**Figure 2:**
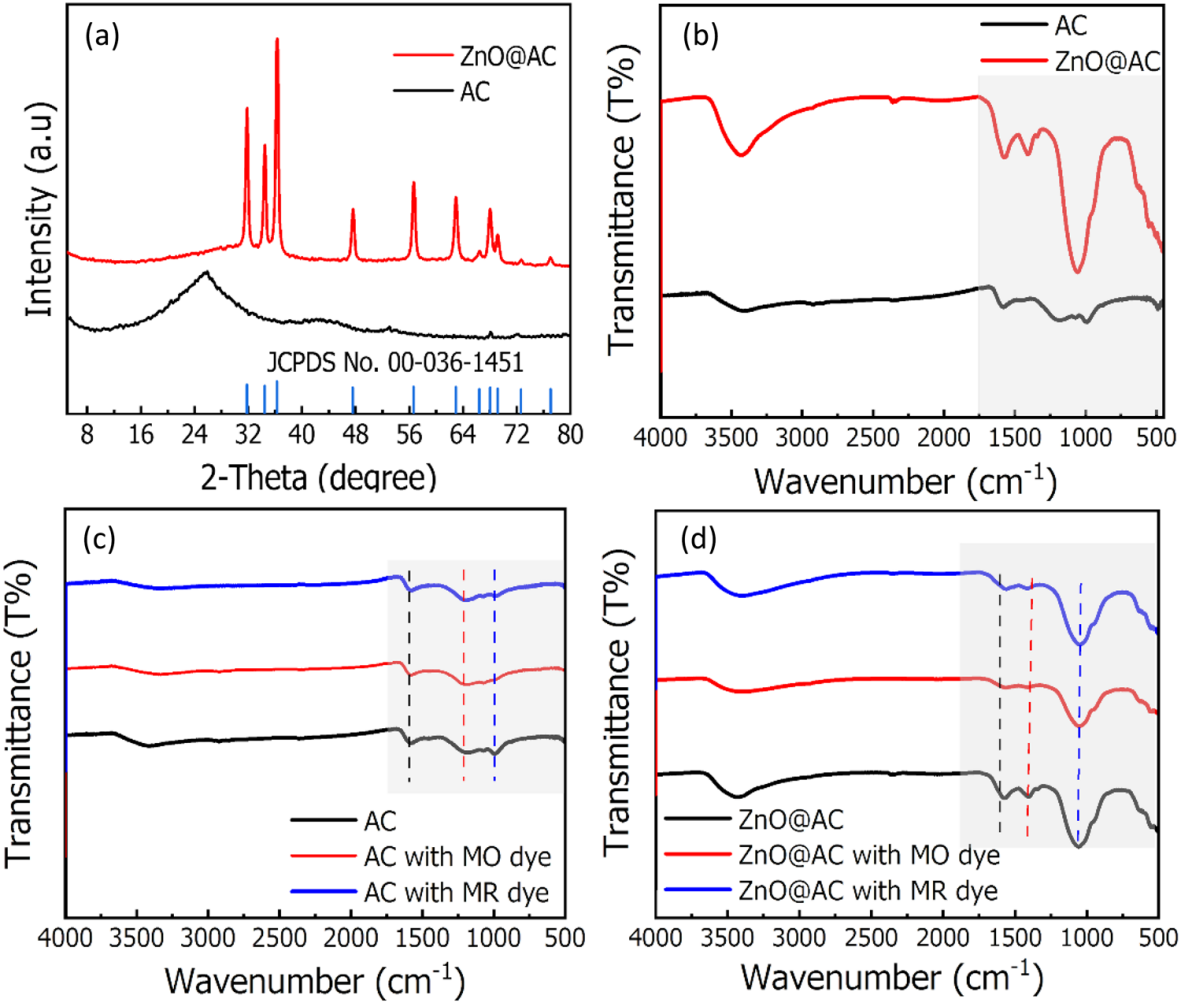
(**a**) XRD patterns of AC and ZnO@AC composite, and (**b**–**d**) FTIR spectra of the AC and ZnO@AC composite with/without MR and MO dyes adsorption.

The FTIR spectra of the AC and ZnO@AC composite with and without MR and MO adsorption were recorded and presented in Fig. 2b–d. The spectra of the AC and ZnO@AC composite in Fig. 2b showed a broad peak at ≈ 3400 cm^−1^ associated with the bands of the O–H groups of the vibration of adsorbed water molecules. Furthermore, the beak of the ZnO@AC composite is stronger than that of the AC peak, indicating the presence of more OH groups, which can play an important role in enhancing the adsorption behavior of the ZnO@AC composite. Furthermore, the AC and ZnO@AC composite displayed small peaks located at 1585 cm^−1^ assigned to C = C groups in carbon material. The weak band located at 972 cm^−1^ in AC is assigned to the C–O groups. FTIR spectra of the ZnO@AC composite displayed two peaks at 1444 and 1062 cm^−1^. The weak peak at 1444 cm^−1^ is assigned for the C-H asymmetric and symmetric bending vibrations, while the strong one can be attributed to Zn–O bonds. The small bands between 400 and 1062 cm^−1^ prove that the ZnO absorption band has a stretching mode of Zn–O, corresponds to the hexagonal ZnO crystal structure^25^. From the FTIR spectrum of the AC loaded with MR and MO dye molecules (Fig. 2c), almost all bands of AC were observed. However, the O–H band at 3500 cm^−1^ of pure AC became lower after dye adsorption, that indicates the formation of bonds between AC and dye molecules. In the ZnO@AC composite spectra after adsorption (Fig. 2d), the intensity of bands at 3500 and 1444 cm^−1^ were displayed, confirming successful dye adsorption.

**Figure 3:**
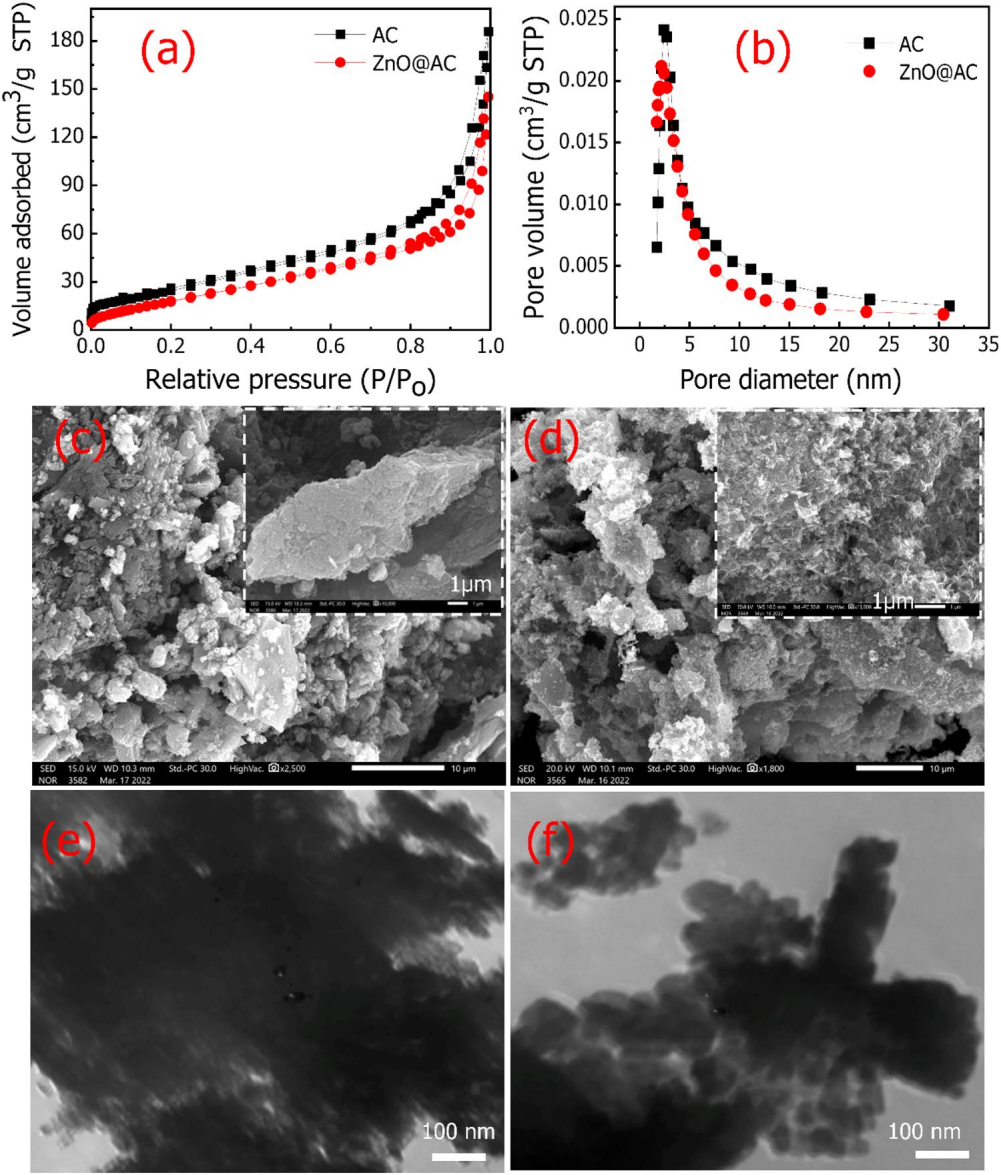
(**a**) Nitrogen adsorption–desorption isotherms, and (**b**) pore volume distribution of the AC and ZnO@AC composite. SEM images of (**c**) AC and (**d**) ZnO@AC composite. TEM images of (**e**) AC and (**f**) ZnO@AC composite.

Besides the functional groups, the specific surface area is an important factor in determining the adsorptive behavior. Nitrogen adsorption–desorption isotherms were utilized to evaluate the specific surface area (S_BET_) and pore structures of the AC and ZnO@AC composite. N_2_ adsorption isotherms of the AC and ZnO@AC composite in Fig. 3a displayed IV type with H_3_ hysteresis loop, which is characteristic of mesoporous materials as stated by the IUPAC categorization^26^. Furthermore, the isotherm is convex upward in the low P/P_0_ region and rises rapidly in the higher P/P_0_ region due to the capillary condensation of mesoporous solids following multi-layer adsorption with a hysteresis loop at P/P_0_ > 0.4, which suggests the presence of mesopores^27^. Additionally, the pore size distribution curves in Fig. 3b showed that mesopore and micropore concentrations are concentrated. By utilizing the BET equation in the pressure range of applicability (P/P_o_ = 0.05–0.30), the S_BET_ and the pore volume of the AC and ZnO@AC composite are estimated to be 76.27 m^2^/g, 0.295 cm^3^/g, and 60.96 m^2^/g; 0.239 cm^3^/g, respectively. In addition, the pore sizes of the AC and ZnO@AC composite are 10.36 and 9.39 nm, respectively. The surface area of our prepared AC, and ZnO@AC are much better than of the pine sawdust derived AC reported by Kalak et al.^28^ The reduction in S_BET_ and pore volume of the ZnO@AC composite compared to those of AC can be attributed to the high rate of agglomeration during the growth of ZnO nanoparticles. This phenomenon agrees with the previous litertutres^29,30^.

The morphologies of the AC and ZnO@AC composite were analyzed via SEM and TEM techniques. The SEM images of the AC (Fig. 3c) and ZnO@AC composite (Fig. 3d) displayed porous structures, which further confirmed the N_2_ adsorption isotherms. As shown in Fig. 3e, the TEM image of AC revealed a highly mesoporous structure, while the TEM image of the ZnO@AC composite in Fig. 3f displayed a near spherical morphology representing ZnO surrounded by an amorphous carbon layer.

**Figure 4:**
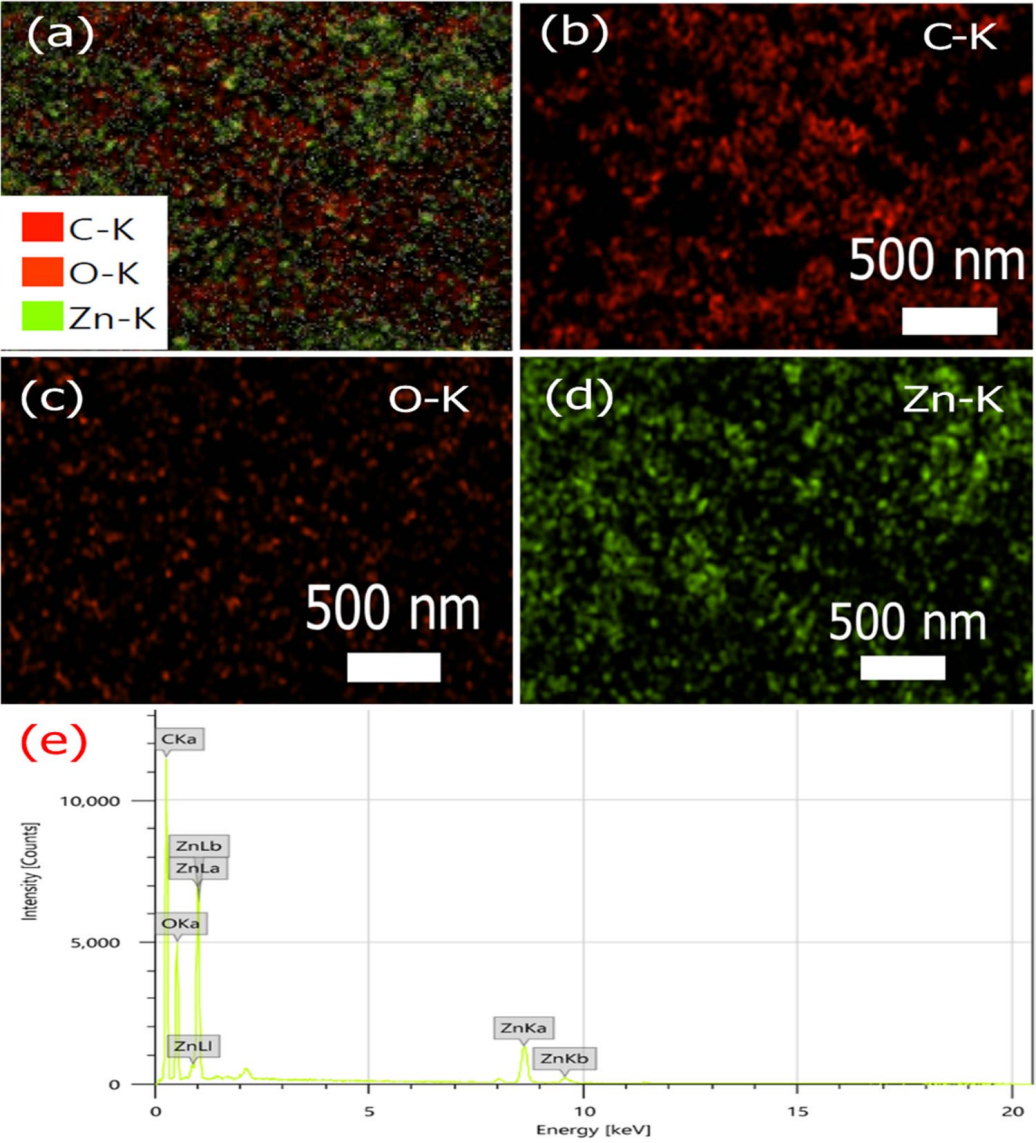
EDS element mapping analysis and EDS spectrum of ZnO@AC composite: (**a**) whole area, (**b**) C–K, (**c**) O–K, (**d**) Zn–K, and (**e**) EDX spectrum.

The energy dispersive X-ray spectroscopy (EDS) and element mapping analysis of the ZnO@AC composite in Fig. 4 display a homogenous distribution of elemental carbon (C), zinc (Zn), and oxygen (O). The presence of the O element may be caused by the incorporation of oxygen, which agrees with the XRD data.

## Adsorption studies

### ***Adsorption capacities of the adsorbent materials*** 

**Figure 5:**
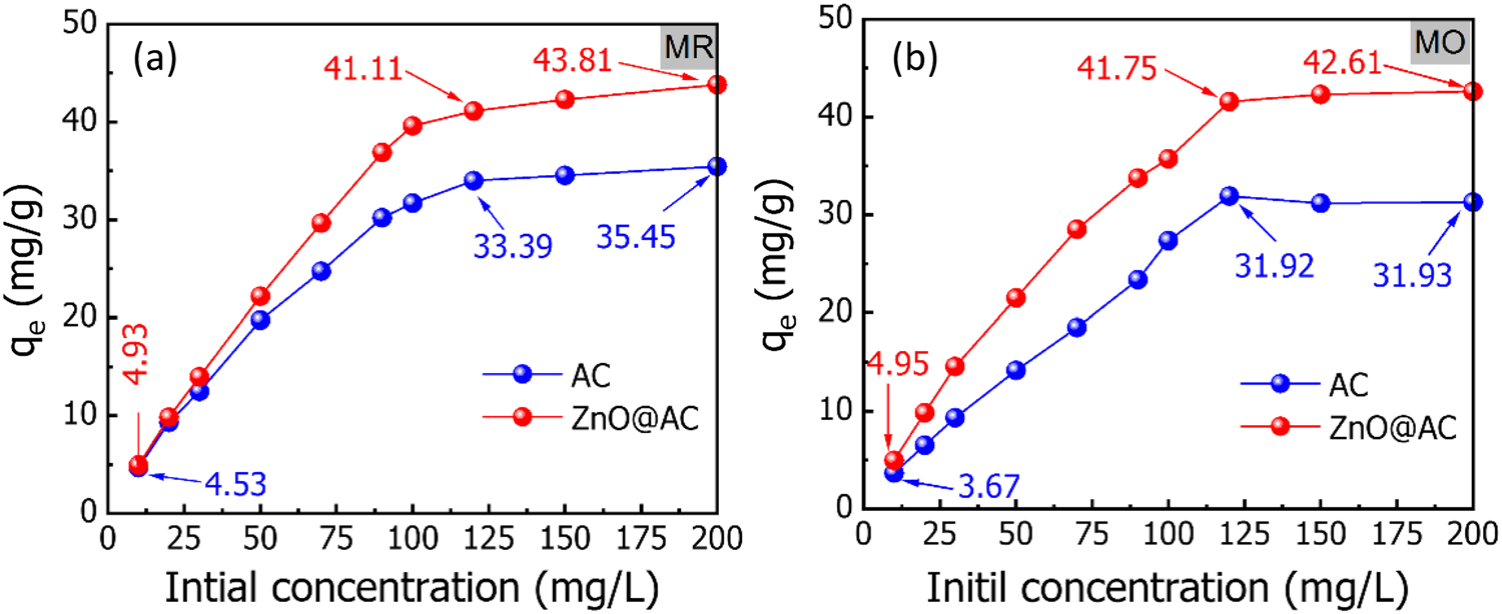
Effect of the initial concentration of (**a**) MR and (**b**) MO dyes on the adsorption capacity (contact time = 60 min; pH = 3; temperature = 20 °C; initial dye concentration = 10–200 mg/L; adsorbent dose = 1.2 g/L).

The adsorption capacity of the AC and ZnO@AC composite were performed by stirring 0.03 g of each adsorbent with 25 mL MR, and MO dyes with different initial concentrations (10–200 mg/L). The pH of all dye solutions was adjusted at 3, and the mixtures were stirred for 60 min at temperature of 20 °C. The obtained data is plotted in Fig. 5. As presented in Fig. 5a, the adsorption capacities of MR and MO are gradually enhanced with increasing the initial MR concentration, followed by a slight increase, and finally a barely noticeable increase. The adsorption capacity of MR was increased from 4.53 to 35.45 mg/g for the AC and from 4.93 to 41.11 mg/g for the ZnO@AC composite by increasing the initial concentration from 10 to 120 mg/L. This is mostly explained by the increase in repulsion forces between the MR molecules on the surface of the adsorbent and the bulk phase following initial adsorption. A further increasing the initial concentration from 120 to 200 mg/L, the adsorption capacities displayed no noticeable enhancement. The maximum adsorption capacity of MR onto the ZnO@AC composite is 43.81 mg/g, which is higher than that of AC (35.45 mg/g). In addition, Fig. 5b showed that by increasing the initial MO concentration from 10 to 120 mg/L, the adsorption capacities gradually increased from 3.67 to 31.92 mg/g for the AC and from 4.95 to 41.57 mg/g for the ZnO@AC composite, followed by a slight increase until 200 mg/L. From the above discussions, we can state two key points: (i) The increasing adsorption capacity by increasing the initial dye concentration is mainly due to enhancing the mass transfer driving force via several collisions between dye molecules and the surface of the adsorbent^24^. This is explained by the increasing repulsion forces between dye molecules on the surface of the adsorbent and the bulk phase following initial adsorption, and (ii) the adsorption capacity of the ZnO@AC composite toward MR and MO are generally higher than that achieved by AC. This can be attributed to increasing the adsorption active sites in ZnO, which is critical for the adsorption process.

### Effect of pH

**Figure 6:**
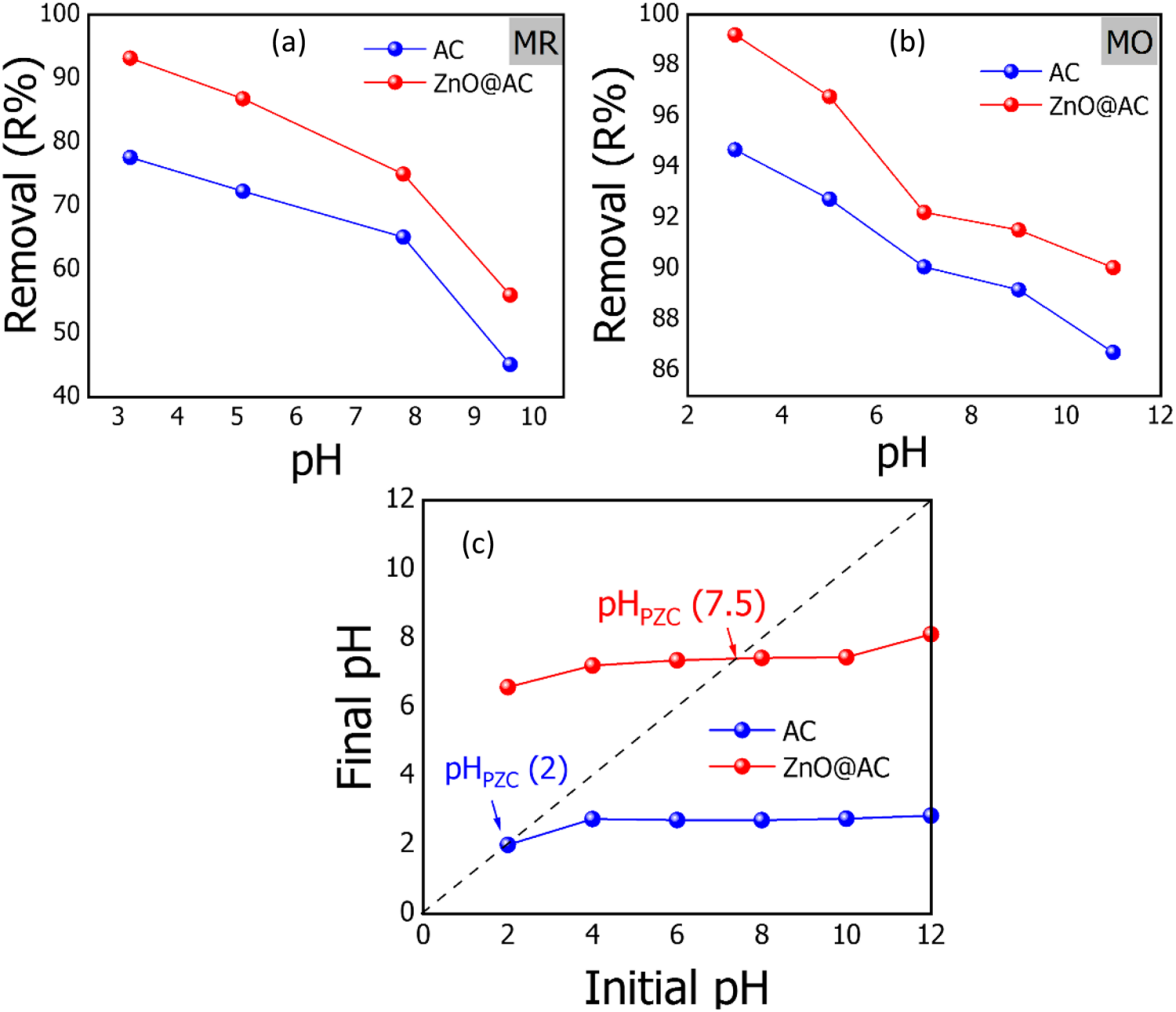
Effect of (**a**, **b**) solution pH (temperature = 20 °C; initial dye concentration = 50 mg/L; adsorbent dose = 1.2 g/L; contact time = 45 min; pH = 3–11) on the removal of MR and MO dye from aqueous solution, and (**c**) the pH_PZC_ of the AC and ZnO@AC composite at 20 ± 2 °C.

The pH of the solution plays a critical role in the adsorption of organic dyes, as the pH change mainly affects the charge of the adsorbent surface and the ionization of the adsorbate^31^. To determine the optimum pH for MR and MO removal by the AC and ZnO@AC composite, the equilibrium adsorption of dyes with an initial concentration of 50 mg/L was investigated in a pH range of 3 to 11. To each dye solution, 0.03 g of adsorbent was added, then the mixtures were kept under magnetic stirrer for 45 min, at 20 °C. As presented in Fig. 6a,b, increasing the initial pH of MR and MO dye solutions leads to a decrease in the overall removal percentage. The highest removal of dyes was achieved at pH 3, and the removal percentages were 77.61 and 93.21% for MR and 94.68 and 99.20% for MO onto AC and ZnO@AC composite, respectively. This behavior could be attributed to the adsorption of dye molecules onto AC, and the ZnO@AC composite was driven by the electrostatic attraction between the adsorbed H^+^ groups and the anionic dye. In acidic conditions (i.e., high H^+^ charges), the adsorbent materials are positively charged, causing remarkable electrostatic interaction with the anionic dye molecules, and thus achieving higher adsorption efficiency^32^. The reducing adsorption in alkaline conditions (i.e., excess OH^–^ ions) can be attributed to the high competition between adsorption sites on the surface of the adsorbent due to the presence of excess OH^–^ ions in the aqueous solution and the anionic groups of the MR and MO dyes on the available adsorption sites^33^. These findings are in agreement with the previous literature^8^.

#### *Point of zero charges (pH*_*PZC*_*)*

The point of zero charge (pH_PZC_) value is quite important to determine the surface charge of the adsorbent, which is usually affected by the pH of the solution. Therefore, pH_PZC_ is used to show the attraction and repulsion forces between the absorbent and adsorbate during the adsorption process. Here, we determine the pH_PZC_ by the pH drift method based on the previous literature^21^. As shown in Fig. 6c, the final pH of systems with AC and ZnO@AC composite increases with increasing the initial pH of the system, indicating the presence of positive surface charges. The pH_PZC_ can be determined by the point of intersection of the final pH line and the initial pH line. Accordingly, the estimated pH_PZC_ values are 2 and 7.5 for the AC and the ZnO@AC composite, respectively. At pH values below pH_PZC_, the surfaces of the AC and ZnO@AC composite were positive, while at pH values above pH_PZC_, the surfaces were negative^34^.

### ***Effect of contact time*** 

**Figure 7:**
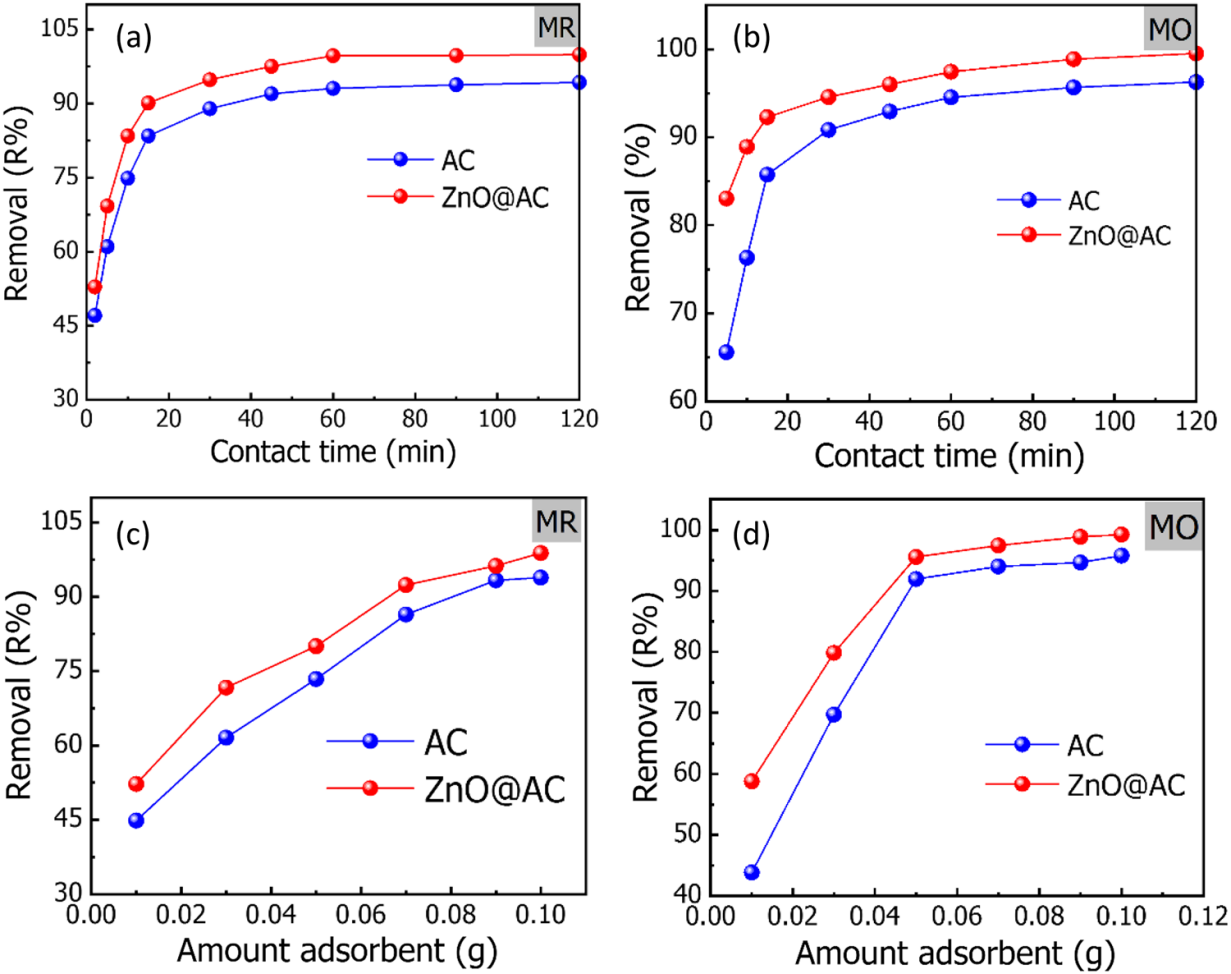
Effect of contact time (pH = 3; temperature = 20 °C; initial dye concentration = 50 mg/L; adsorbent dose = 1.2 g/L; contact time = 2–120 min) on the removal of (**a**) MR and (**b**) MO dyes from aqueous solution. Effect of adsorbent dose (pH = 3; temperature = 20 °C; initial dye concentration = 50 mg/L; adsorbent dose = 0.4–4 g/L; contact time 60 = min) on the removal of (**c**) MR and (**d**) MO dyes from aqueous solution.

The contact time between absorbent and adsorbate is critical in designing the systems for wastewater treatment. Thus, the influence of contact time on the adsorption efficiency of MR and MO dyes onto the AC and ZnO@AC composite was investigated by using dye solutions with an initial concentration of 50 mg/L, the acidity of the dye solutions was adjusted to a pH of 3. To each 25 mL dye solution, 0.03 g of adsorbent was added and stirred various times, ranging from 2 to 120 min at room temperature (20 °C). The adsorption efficiency of MR and MO dyes is significantly improved by increasing the stirring time from 2 to 60 min. As presented in Fig. 7a, the removal percentage of MR increased from 47.10 to 93.97% on AC and from 52.86 to 99.69% on the ZnO@AC composite. Furthermore, Fig. 7b showed that the removal percentage of MO was increased from 65.58 to 94.59% for AC and from 83.03 to 97.43% for the ZnO@AC composite. The higher adsorption at the early step (i.e., until 60 min) can be assigned to the presence of more unoccupied adsorption sites. In the later stage, an adsorption plateau has been observed due to the interactions between the dye molecules on the adsorbent, and the bulk phase has become more and more repulsive^35^.

### Effect of adsorbent dosage

The adsorbent dose is essential to avoiding wasting the adsorbent materials after equilibrium^8^. In our study, various weights of adsorbent ranging from 0.01 to 0.1 g were mixed separately with a 25 mL dye solution with an initial concentration of 50 mg/L, the acidity of the dye solutions was adjusted to be at a pH of 3, and all mixtures were stirred for 60 min at room temperature (20 °C). As presented in Fig. 7c,d, by increasing the weight of adsorbents from 0.01 to 0.1 g, the removal percentage of MR and MO dyes gradually increased, which was mainly cold be assigned to the more active sites viable for the interaction with the dye molecules.

### ***Adsorption kinetics***

Adsorption kinetics analysis was performed to investigate and comprehend the adsorption process and rate. Here, nonlinear regression kinetic models including pseudo-first-order, pseudo-second-order, intra-particle diffusion, and Elovich models were applied to investigate the adsorption behavior of both dyes onto the AC and ZnO@AC composite. The pseudo-first order model is expressed by Eq. (4)^36^

4 $$q_{t} = q_{e} \left( {1 - e^{{ - k_{1} t }} } \right)$$

where q_e_ and q_t_ are the adsorbed dyes (mg/g) at equilibrium and at a time t (min), respectively. k_1_ is the rate constant (min^−1^).

Moreover, the pseudo-second-order model is expressed by Eq. (5)

5 $${\text{q}}_{t} = \frac{{q_{e}^{2} k_{2} t}}{{1 + k_{2} q_{e} t}}$$

where k_2_ is the pseudo-second-order rate constant (g/mg min), q_e_ and q_t_ are the same as in Eq. (4).

**Figure 8:**
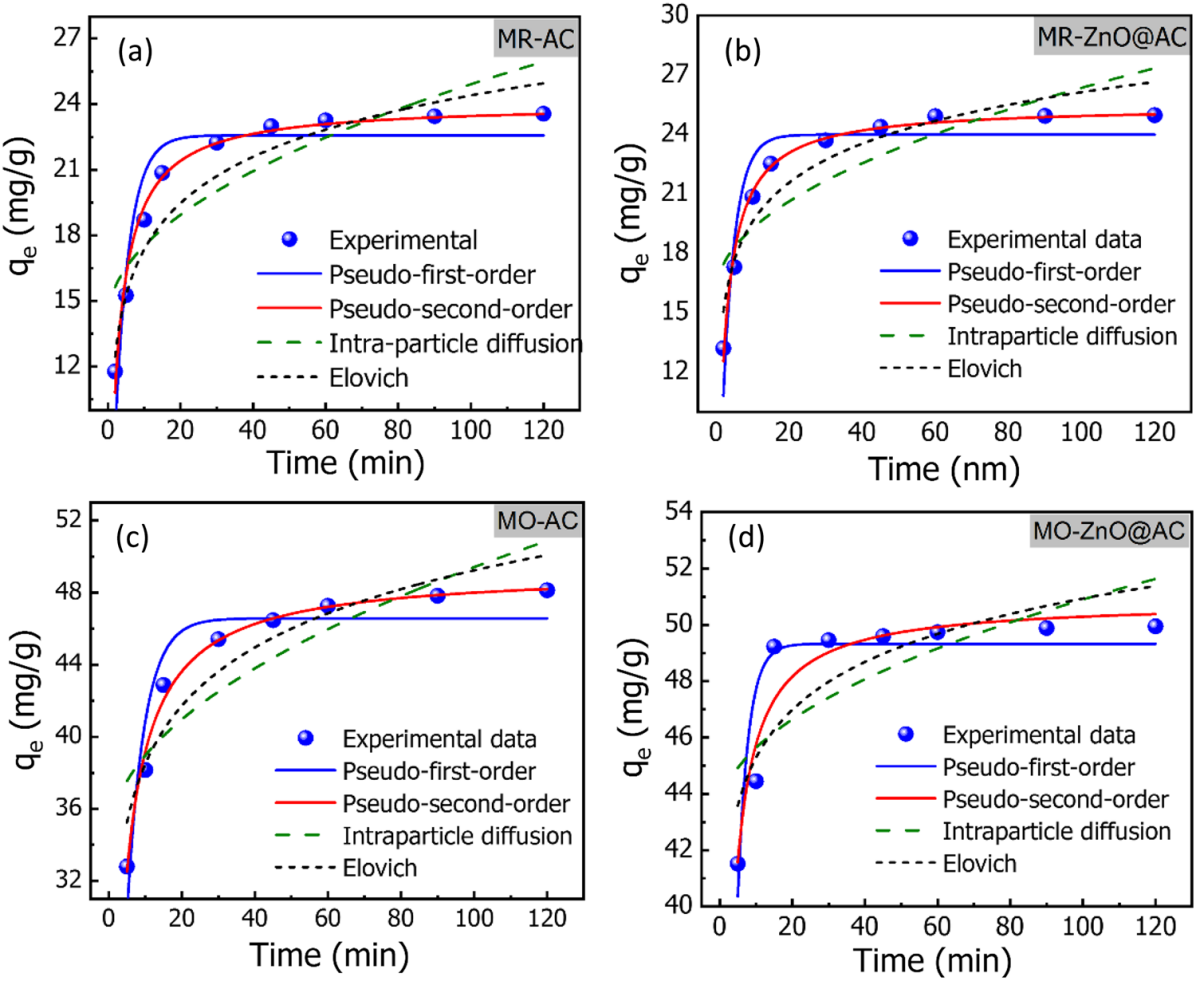
Experimental and kinetic adsorption models for adsorption of MR and MO onto the AC and ZnO@AC composite (pH = 3; temperature = 20 °C; initial dye concentration = 50 mg/L; adsorbent dose = 1.2 g/L; contact time = 2–120 min).

As plotted in Fig. 8a–d, the adsorption kinetics of MR and MO dyes onto the AC and ZnO@AC composite began quickly, increased a little, and then plateaued after 60 min. From the estimated kinetic parameters in Table 1, the correlation coefficient value (R^2^) for the pseudo-second-order kinetic model is near one and higher than the first-order model. This indicates that the pseudo-second-order adsorption model is more appropriate than the pseudo-first-order model for the adsorption of the two kinds of dyes. As reported in the literature, the pseudo-first-order kinetic model represents the physisorption process, while the pseudo-second-order kinetic model describes the chemisorption process^37^. Therefore, the kinetic data demonstrate that MR and MO adsorption onto the AC and ZnO@AC composite adsorbent materials happens by chemisorption, such as electron exchange between the adsorbent and the adsorbate^37^. The estimated q_e_ values from the pseudo-second-order models for the adsorption of MR onto the AC and ZnO@AC composite are 24.030 and 25.442 mg/g, respectively. Moreover, the q_e_ values are 49.573 and 50.853 mg/g for the adsorption of MO onto the AC and ZnO@AC composite, respectively. Despite the adsorption of MR and MO dyes fitted with the pseudo-second-order model, the rate-limiting step is difficult to determine by the pseudo-first-order or the second-order models. Thus, the Weber–Morrison (ID-WM) model, which describes the intraparticle diffusion model was applied to investigate whether the process was controlled by film diffusion (the movement of ions from the bulk solution to the external surface of the adsorbent) or intraparticle diffusion (movement of ions into the interior of the adsorbent)^38^. The non-linear form of ID-WM is expressed by Eq. (6)^39^

6 $$q_{t} = k_{diff} t^{0.5} + C$$

where k_diff_ is the intraparticle diffusion rate constant (mg/g min^1/2^) and C (mg/g) is the boundary layer thickness.

**Table 1:** The kinetic parameters for MR and MO adsorption by the AC and ZnO@AC composite were obtained from the non-linear models.

Kinetic model	Parameter	AC		ZnO@AC composite	
		MR	MO	MR	MO
Pseudo-first order	qe (mg/g)	22.574 ± 0.642	46.234 ± 0.316	23.99 ± 0.599	49.329 ± 0.590
	K_1_ (min^−1^)	0.253 ± 0.038	0.210 ± 0.021	0.299 ± 0.042	0.341 ± 0.034
	R^2^	0.862	0.877	0.868	0.789
Pseudo-second order	qe (mg/g)	24.030 ± 0.289	49.573 ± 0.826	25.442 ± 0.205	50.853 ± 0.514
	K_2_ (g/mg. min)	0.017 ± 0.001	0.008 ± 0.004	0.020 ± 0.001	0.018 ± 0.003
	R^2^	0.982	0.989	0.998	0.905
Intra-particle diffusion	K_diff_ (mg/g min^0.5^)	1.081 ± 0.253	1.529 ± 0.367	1.036 ± 0.260	0.770 ± 0.280
	C (mg/g)	14.102 ± 1.639	34.129 ± 2.509	15.990 ± 1.683	43.200 ± 1.918
	R^2^	0.683	0.701	0.651	0.484
	α (mg/g h)	87.526	286.826	1776.277	2.493*10^7^
Elovich model	β (g/mg)	0.326	0.352	0.214	0.407
	R^2^	0.945	0.903	0.898	0.735

pH = 3; temperature = 20 °C; initial dye concentration = 50 mg/L; adsorbent dose = 1.2 g/L; contact time = 2–120 min.

As presented in Fig. 8a–d and the data in Table 1, the intra-particle diffusion model for the adsorption of MR and MO onto AC and the ZnO@AC composite showed the least agreement with the experimental data. Thus, the entire adsorption process may be controlled using external mass transfer and intraparticle diffusion. It can be inferred from the explanation above that the pseudo-second-order model is the one that best fits the experimental results^40^. Elovich is another kinetic model, that is satisfied for the chemisorption process and for the heterogeneous adsorbent surface, hence modeling many dye adsorption systems^41^. The non-linear form of the Elovich model can expressed by Eq. (7).

7 $$q_{t} = \frac{1}{\beta }\ln \left( {1 + \alpha \beta t} \right)$$

where q_t_ is the amount of absorbed or released adsorbate in time t, α is the rate of initial adsorption (mg/g.min) and β is the desorption constant (g/mg) related to the extent of surface coverage and the activation energy for chemisorption.

As shown in Table 1, Elovich parameters suggest that the initial constant rate (α) is greater than the desorption coefficient (β) for AC and ZnO@AC composite, which indicates that the adsorption process is governed by the chemisorption^42^. In addition, the higher R^2^ value of the Elovich model than that of the intraparticle diffusion model suggests the Elovich model is most appropriate, thus confirming the chemisorption process involving the exchange of electrons between the adsorbent and adsorbate is likely the rate-limiting step^43^. All kinetic finding indicate that the adsorption of MR and MO dyes onto AC and ZnO@AC composite is controlled by chemisorption.

### ***Adsorption isotherms***

The Langmuir and Freundlich isotherm models are frequently used to describe solid–liquid adsorption systems. A monolayer adsorption onto a uniform surface with a finite number of identical sites is described by the Langmuir model. The non-linear relation of this model can be expressed by Eq. (8)^44^

8 $$q_{e} \frac{{q_{max} K_{L} C_{e} }}{{1 + K_{L} C_{e} }}$$

where q_max_ (mg/g) is the maximum adsorbate, and K_L_ (L/mg) is the Langmuir constant.

The Freundlich model is usually used to explain heterogeneous surfaces and multilayer adsorption systems, the non-linear form of Freundlich is given by Eq. (9)^39^.

9 $$q_{e} = K_{f} C_{e}^{1/n}$$

where *K*_f_ (L/g) and *n* are the Freundlich constants, which represent the adsorption capacity and intensity, respectively.

**Figure 9:**
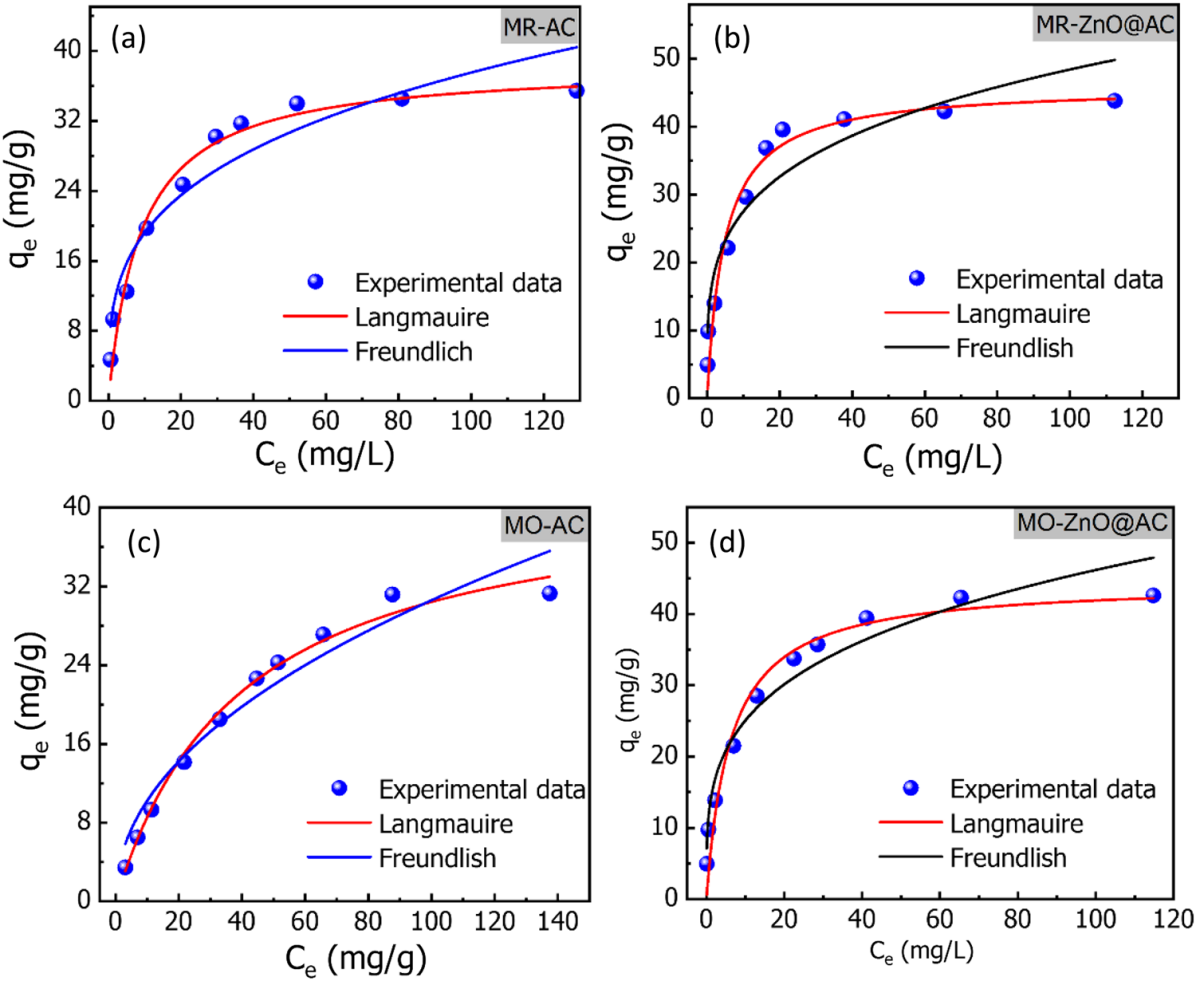
The experimental Langmuir, and Freundlich isotherm models for MR and MO adsorption onto the AC and ZnO@AC composite (pH = 3; temperature = 20 °C; initial dye concentration = 10–200 mg/L; adsorbent dose = 1.2 g/L; contact time = 45 min).

To validate the adsorption isotherm models, the Chi-square (*χ*^2^) error parameter was evaluated in addition to R^2^. The plots of isotherm models are shown in Fig. 9a–d, and their related parameters are given in Table 2. Based on greater R^2^ and lower *χ*^2^ values, the experimental results of the adsorption of MR and MO dyes onto the AC and ZnO@AC composite are fitted by Langmuir model. This indicates the formation of dye monolayer on the surface with homogenous localized adsorption sites^22^. The interaction between an adsorbate and an absorbent is typically described by the Langmuir constant K_L_. The values of K_L_ for MR are 0.112 and 0.208 L/mg, and for MO, they are 0.210 and 0.261 L/mg for the AC and ZnO@AC composite, respectively. The higher K_L_ for MR and MO on to ZnO@AC composite demonstrates more stable interactions than interactions with AC. The maximum monolayer adsorption capacities for MR onto the AC and ZnO@AC composite are 38.408 and 45.992 mg/g, respectively. Moreover, the maximum monolayer adsorption capacity of MO onto AC and ZnO@AC composite are 42.508 and 44.500 mg/g, respectively. The higher adsorption capacity of ZnO@AC composite can be attributed to the higher surface area and the presence of ZnO nanoparticles, which act as active centers for adsorption. The maximum adsorption capabilities of MR on the AC and ZnO@AC composite are 38.408 and 45.992 mg/g, respectively. In addition, the values of MO onto AC and ZnO@AC composite are 42.508 and 44.500 mg/g, respectively. The higher adsorption capacities of the ZnO@AC composite can be assigned due to the presence of ZnO nanoparticles, which serve as active centers for adsorption, as well as the higher surface area as mentioned in the previous section (Fig. 3a).

**Table 2:** Adsorption isotherm parameters for MR and MO dye adsorption onto AC and ZnO@AC composite estimated from the non-linear models.

Isotherm model	Parameter	AC		ZnO@AC composite	
		MR	MO	MR	MO
Langmuir	q_m_ (mg/g)	38.408 ± 1.659	42.508 ± 1.919	45.992 ± 2.316	44.500 ± 2.356
	K_L_ (L/mg)	0.112 ± 0.020	0.210 ± 0.021	0.208 ± 0.048	0.261 ± 0.039
	Adj. R^2^	0.968	0.990	0.953	0.951
	*X*^2^	4.202	1.069	10.161	9.001
Freundlich	K_f_ (L/g)	9.828 ± 1.502	3.417 ± 0.743	15.649 ± 2.109	13.601 ± 1.377
	N	3.45 ± 0.039	2.101 ± 0.051	4.082 ± 0.0368	3.77 ± 0.027
	Adj. R^2^	0.914	0.940	0.896	0.933
	*X*^2^	11.308	6.151	22.397	9.262

pH = 3; temperature = 20 °C; initial dye concentration = 10–200 mg/L; adsorbent dose = 1.2 g/L; contact time = 45 min.

### Effect of temperature and thermodynamic analysis

**Figure 10:**
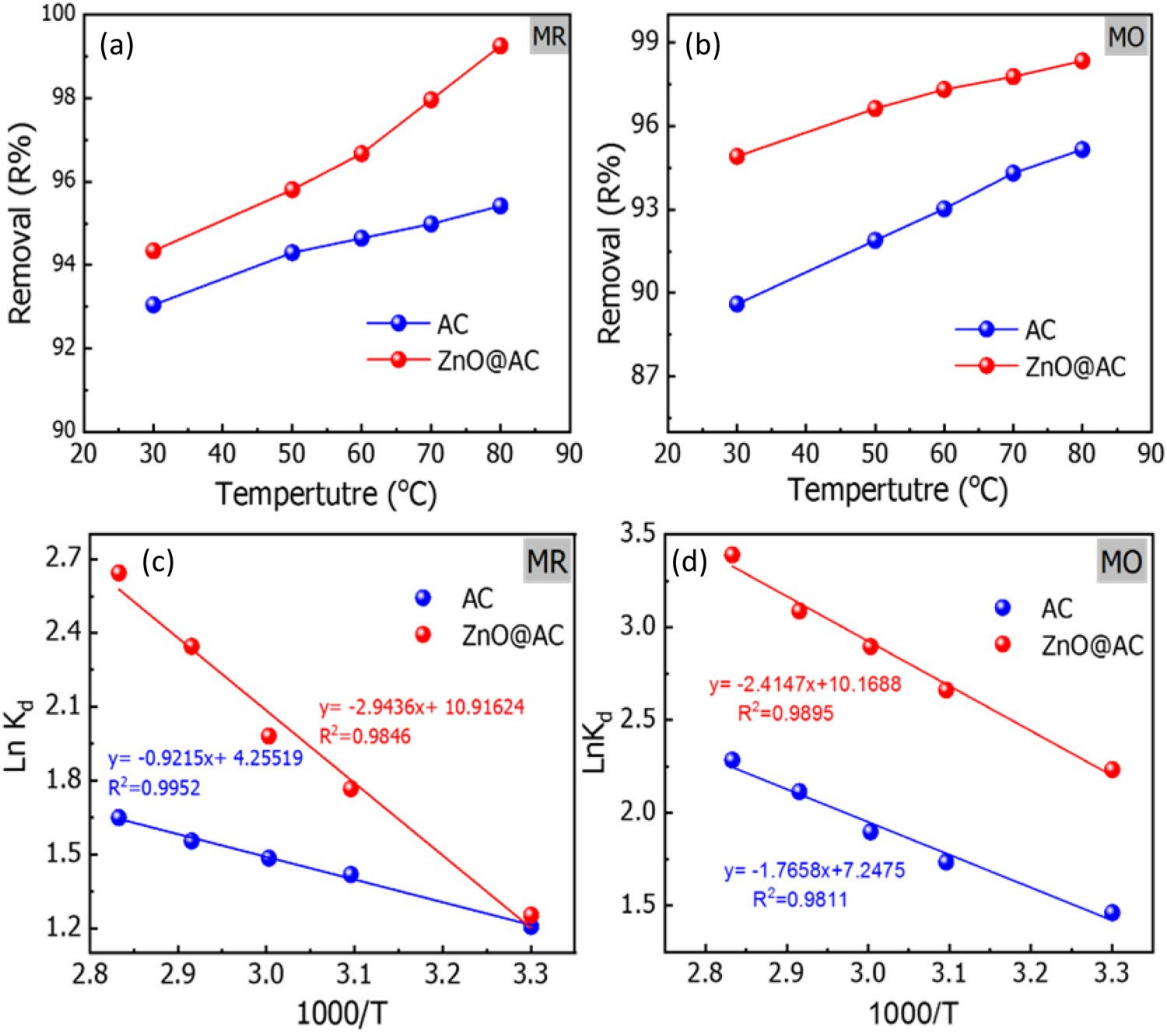
Effect of temperature (pH = 3; temperature = 20 °C; initial dye concentration = 50 mg/L; adsorbent dose = 1.2 g/L; contact time = 60 min; temperature = 30–80 °C) on the removal of MR and MO dyes (**a**, **b**), and Van’t Hoff plots of MR and MO adsorption onto AC and ZnO@AC composite (**c**, **d**).

The impact of temperature on the elimination of MR and MO dyes by AC and ZnO@AC composite was examined. The equilibrium adsorption experiments were conducted at five distinct temperatures within a range of 30–80 °C. According to the results in Fig. 10a,b, the removal efficiency of MR and MO dyes is enhanced by increasing the temperature from 30 to 80 °C. This indicates that the adsorption mechanism is endothermic^45^. The related thermodynamic parameters, such as the change in Gibbs free energy ($\Delta {\text{G}}^{ \circ }$), enthalpy ($\Delta {\text{H}}^{ \circ }$), and entropy ($\Delta {\text{S}}^{ \circ }$) were estimated at different temperatures (303–353 K) by applying Van't Hoff plots^45^. This parameter could be used to explore either exothermic or endothermic spontaneous adsorption activities, as well as the degree of the disorder.

10 $$\ln {\text{K}}_{{\text{d}}} = \frac{{\Delta {\text{S}}^{ \circ } }}{{\text{R}}} - \frac{{\Delta {\text{H}}^{ \circ } }}{{{\text{RT}}}}$$

11 $$\Delta {\text{G}}^{ \circ } = \Delta {\text{H}}^{ \circ } - {\text{T}}\Delta {\text{S}}^{ \circ }$$

12 $$\Delta {\text{G}}^{ \circ } = - {\text{RT}}\ln {\text{K}}_{{\text{d }}}$$

where ΔS° (J mol^−1^ K^−1^), ΔG° (kJmol^−1^), and ΔH° (kJmol^−1^) represent the changes in entropy, Gibbs free energy, and enthalpy, respectively. T is the adsorption temperature (Kelvin), R is the gas constant (8.3145 J mol^−1^ K^−1^), and K_d_ (Q_e_/C_e_) is the change in kinetic energy.

The numerical values of ΔH°, ΔS°, and ΔG° were estimated from the slope and intercept of the linear Van't Hoff plots of ln K_d_ versus 1/T (Fig. 10c,d).

The values of ∆G° were calculated at different temperatures according to Eq. (12). The values of ΔH°, ΔS°, and ΔG° are in Table 3. The negative values of ΔG° for the MR and MO adsorption processes suggest the adsorption is spontaneously and thermodynamically favorable^46^. In contrast, the positive ΔS° values indicate an increase in the randomness and disorder at the liquid–solid interface during the adsorption^47^. The positive values of ΔH° denote the endothermic nature of the adsorption process. Suggesting an increase in temperature enhances the adsorption performance as heating the active sites of adsorbents strengthens the bonds between the adsorbate molecules. This also confirms that the adsorption is chemically processed. In addition, the ΔH° values of MR and MO adsorption by the ZnO@AC composite are greater than those for adsorption by AC. This indicates a stronger interaction of MR and MO dyes with the ZnO@AC composite^48^.

**Table 3:** Thermodynamic parameters for MR and MO adsorption by AC and ZnO@AC composite.

Parameter	AC		ZnO@AC composite	
	MR	MO	MR	MO
ΔH° (Kj/mol)	7.6614	14.681	24.473	20.101
ΔS° (J/ mol. K)	35.378	60.256	90.758	84.543

Temperature (K)	ΔG° (kJ/mol)			
	MR	MO	MR	MO
303	− 3.04	− 3.68	− 3.16	− 5.62
323	− 3.81	− 4.66	− 4.75	− 7.15
333	− 4.11	− 5.25	− 5.49	− 8.02
343	− 4.44	− 6.03	− 6.69	− 8.80
353	− 4.84	− 6.70	− 7.76	− 9.95

pH = 3; temperature = 20 °C; initial dye concentration = 50 mg/L; adsorbent dose = 1.2 g/L; contact time = 60 min; temperature = 30–80 °C.

### Selective adsorption of AC and ZnO@AC composite

**Figure 11:**
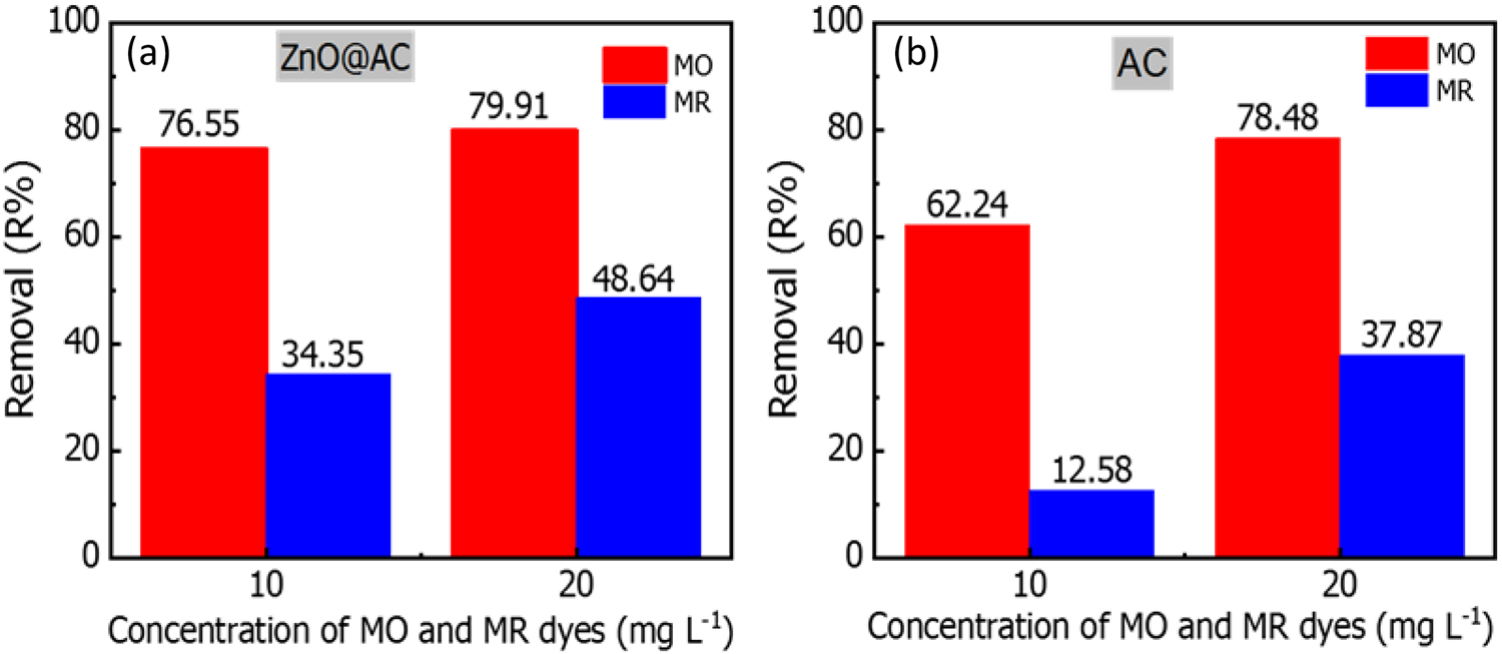
The adsorption selectivity of (**a**) the ZnO@AC composite, and (**b**) the AC toward MO and MR dyes in binary solution (temperature = 20 °C; adsorbent dose = 2 g/L; contact time = 30 min).

To evaluate the adsorption selectivity of the AC and ZnO@AC composite toward MR and MO dyes, 0.05 g of adsorbent was mixed with 20 mL of MB: MO mixture dye solutions in a 1:1 (v/v) ratio. The initial concentrations of MR and MO dyes are 10 and 20 mg/L, and the adsorbent weight was fixed at 0.05 g. The adsorption was carried out under magnetic stirring at 22 °C for 30 min. Then the adsorbent was separated by centrifugation, and the concentration of dyes residue were recorded by UV–vis spectroscopy. As presented in Fig. 11, ZnO@AC composite, and AC displayed higher affinity toward MO dye in comparison to MR dye. This agrees with the kinetic (Fig. 8 & Table 1) and isotherm (Fig. 9 & Table 2) analysis.

### Regeneration

**Figure 12:**
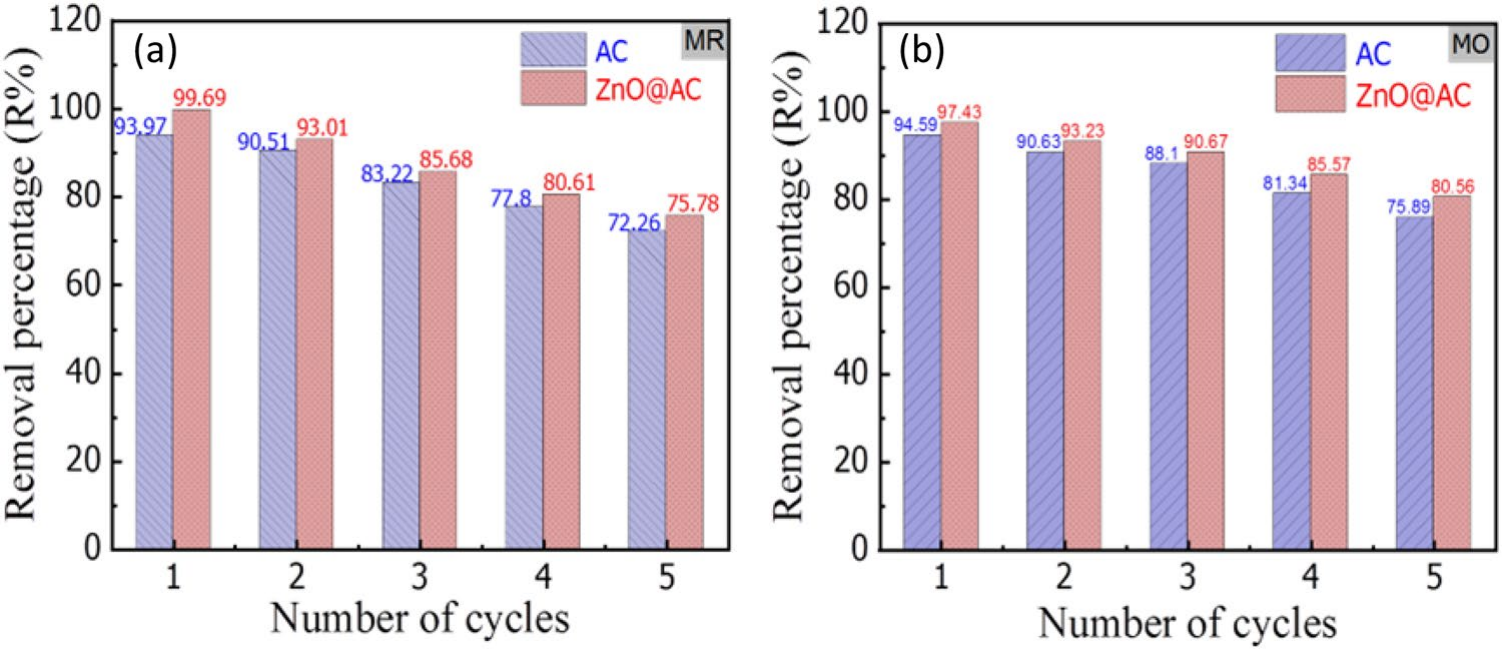
Regeneration of the AC and ZnO@AC composite up to five successive desorption-adsorption cycles.

To perform efficient pollutant sequestration through adsorption, the sorbent should have the ability to be recycled without undergoing a considerable reduction in its adsorption capacity. Multiple uses of adsorbent materials are quite important in reducing the total cost of the treatment process^49^. Here, the reusability of the AC and ZnO@AC composite was investigated by monitoring their adsorption toward MR and MO dyes under ideal conditions for five cycles. First, the adsorption processes of MR and MO onto the AC and ZnO@AC composite were performed at 60 min. Then, the regeneration studies were conducted by sonicating MR-/MO-adsorbed onto AC and ZnO@AC composite in 20 mL ethanol for 30 min at room temperature (20 °C). After desorption, the regenerated AC and ZnO@AC composite were reused for MR and MO dye adsorption, and five cycles of regeneration and adsorption were carried out in succession. Figure 12 displays that both AC and the ZnO@AC composite depict good recyclability after five cycles of desorption-adsorption. As shown in Fig. 12a, the removal percentage of MR decreased by 23.1 and 24% onto AC and ZnO@AC composite, respectively. Moreover, in Fig. 12b, the removal of MO onto the AC and ZnO@AC composite was reduced by 19.8 and 17.3%, respectively. Thus, the AC and ZnO@AC composite are highly reusable for the removal of anionic dyes from wastewater.

### ***Adsorption mechanism***

**Figure 13:**
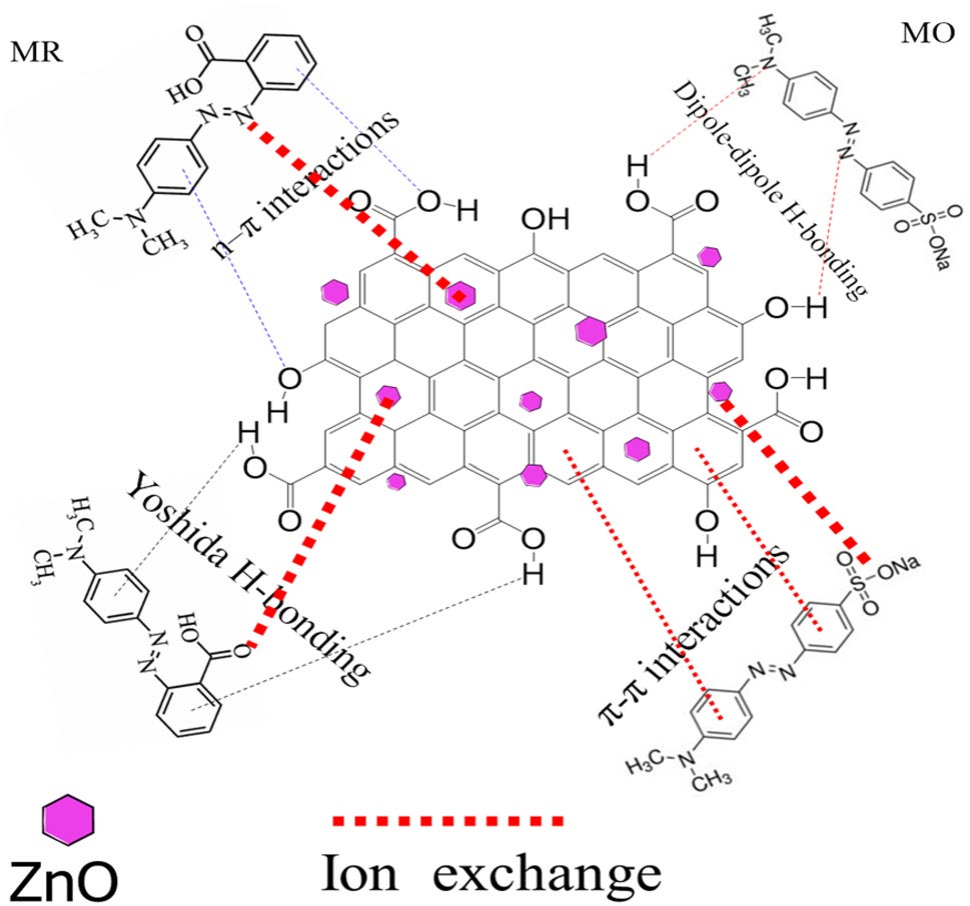
The possible mechanism for adsorption of MR and MO onto ZnO@AC composite.

As discussed above, the adsorption of MR and MO dyes onto AC and ZnO@AC composite is a chemisorption process. The mechanism of adsorption of dyes onto a surface of absorbent is dependent on the nature of both adsorbent and adsorbate and the probable adsorbate/adsorbent interactions. Electrostatic interactions take place in both dye adsorption, which is controlled by the pH of the solution. As discussed in effect of pH, the adsorption of MR and MO steadily declined with increasing pH (Fig. 6a,b). Furthermore, Fig. 6c shows that the pH_ZPC_ of AC and ZnO@AC composite surfaces were performed at pH 2, and pH 7.2. It indicates that the repulsion between the anionic dyes and the positively charged surface occurs in the basic conditions, which reduce the attraction and thus overall adsorption. In the other words, the electrostatic interactions between the negatively charged anionic dyes and positively charged surface adsorbents will occur in acidic conditions^21^. Hydrogen bonding between the hydrogen of the hydroxyl groups (H-donor) on the surface of adsorbent materials with oxygen or nitrogen in dye molecules (H-acceptor) can occur. This kind of H-bonding is called dipole–dipole H-bonding. H-bonding can also be found between the OH groups on the surface of the adsorbent and the aromatic rings in the dye molecules, which is called Yoshida H-bonding. To further confirm the adsorption mechanism, FTIR spectra before and after the adsorption of MR and MO dyes were recorded. By comparing the FTIR of pure adsorbent materials presented in Fig. 2b with adsorbents loaded with dye molecules shown in Fig. 2c,d, it can be observed that the adsorption of MR and MO dyes caused several alterations in the absorption peaks. After absorption of MR and MO onto AC, the absorption peak of the OH groups were diminished, which might be attributed to the formation of H-bonds. This further indicates the presence of dipole–dipole interaction and Yoshida H-bonding. Another factor that can affect the adsorption mechanism is the n–π interaction between the oxygen and nitrogen (electron-donating) on the adsorbent surface and the π-system in the aromatic rings of the dye molecules (electron acceptor). From the FTIR spectrum, the peak located at 972 cm^−1^ for the C–O group was remarkably diminished indicating the presence of an n–π interaction^50^. π–π interactions (π–π electron donor–acceptor interactions) between the π-electrons of carbon and the π-electrons of the adsorbate aromatic rings are another possible factor. For the ZnO@AC composite, the ion exchange between the active site of the dye anion and the Zn ions takes place. To conclude, as presented in Fig. 13, the adsorption mechanism of anion dyes with ZnO@AC composite has been controlled by electrostatic attraction, H-bonding, Yoshida H-bonding, n–π interaction, and ion exchange. However, the limited presence of OH groups in the prepared adsorbents makes H-bonding and n–π interactions not the major factors in the adsorption mechanisms.

### Comparison of adsorbent with other reported adsorbents

According to the literature, several carbon-based materials have been utilized for the removal of MR and MO dyes from aqueous solutions. The competitiveness of our absorbent was examined against the other reported adsorbents, as illustrated in Table 4. Our adsorbents showed competitive adsorption ability and affinity towards MR and MO dyes concerning the other adsorbents. Furthermore, our developed adsorbents are cost-effective for practical applications. Hence, both AC and ZnO@AC composite is highly recommended as efficient adsorbents for anionic dyes due to their easy preparation, effectiveness, and reusability, which are favorable for the treatment of effluents containing dyes, such as textile industry effluents.

**Table 4:** Comparison of adsorption MR and MO dyes by our adsorbents and different adsorbents reported in the literature.

Adsorbent	Dye	pH	q_m_ (mg/g)	References
Magnetic mesoporous-AC (MMAC) from rice husk	MO	3	13.21	^51^
AC from date seeds	MO	8	7.57	^52^
ZnO-AC (ZnO@AC)	MO	–	107.97	^53^
ZnO-orange peel (ZnO@OP)	MO	–	166.25	^53^
ZnO nanoparticles (ZnO-NPs)	MO	6	65.2	^19^
ZnO/polyaniline nanocomposite	MO	2	240.84	^54^
Rayon-based AC fibers (ACFs)	MO	6	700	^55^
AC from coffee waste	MO	3	658	^56^
AC from popcorn	MO	–	969.0	^57^
Pomelo peel-derived porous carbon	MO	3	680.3	^58^
Porous carbon from potassium citrate	MO	2	927	^59^
ZnO@AC composite from wood sawdust	MO	3	42.61	This work
NiO@hyphaene thebaica seed-derived carbon	MR	2.5	129.87	^8^
AC from rosemary root	MR	3.25	154.53	^60^
Carbon from foeniculum vulgare seeds	MR	–	135	^61^
AC from durian seed	MR	6	384.62	^62^
Carbon from biogas plant waste	MR	–	113	^63^
ZnO@AC composite from wood sawdust	MR	3	43.81	This work

## Conclusions

The adsorption of MR and MO onto AC and ZnO@AC composite derived from wood sawdust has been reported. The maximum adsorption of MR, and MO dyes was achieved in the acidic medium at pH of 3 under magnetic stirrer for 60 min. The experimental data were fitted with the Langmuir adsorption model, and the estimated adsorption capacity were 35.45 and 43.81 mg/g for the adsorption of MR onto the AC and ZnO@AC composite, respectively. On the other hand, MO displayed maximum adsorption capacities of 31.93 and 42.61 mg/g onto AC and ZnO@AC composite, respectively. The kinetic studies confirmed that the pseudo-second order kinetic model reveals a chemical adsorption process in which electrostatic attraction and π–π interactions can be used to explain both MR and MO adsorption onto the AC and ZnO@AC composite. In addition, the thermodynamic analysis confirmed that the adsorption process is spontaneous and endothermic. These findings demonstrated the possibility of employing AC and ZnO@AC composite derived from wood sawdust for the removal of color decontamination from wastewater.

### Supplementary Information

Supplementary Information.

## Data Availability

All data generated or analyzed during this study are included in this published article and its supplementary information file.
