# Supplementary material for: ZnO@ activated carbon derived from wood sawdust as adsorbent for removal of methyl red and methyl orange from aqueous solutions
Source: Sci Rep. 2024 Mar 5;14:5384. doi: 10.1038/s41598-024-55158-7 (PMC10915167; doi:10.1038/s41598-024-55158-7)
Supplement: Supplementary file 1 — Supplementary Information. [file 41598_2024_55158_MOESM1_ESM.docx]

Supplementary information

**ZnO@ Activated carbon derived from wood sawdust as adsorbent for removal of methyl red and methyl orange from aqueous** **solutions**

Nessma S. M. Sayed^1^, Abdelaal S. A. Ahmed^1*^, Mohamed H. Abdallah^1^, Gamal A. Gouda^1^

^1^ Chemistry Department, Faculty of Science, Al-Azhar University, Assiut 71524, Egypt

*Corresponding authors [abdelaalsaiyd@gmail.com](mailto:abdelaalsaiyd@gmail.com); [abdelaalsaiyd@azhar.edu.eg](mailto:abdelaalsaiyd@azhar.edu.eg)


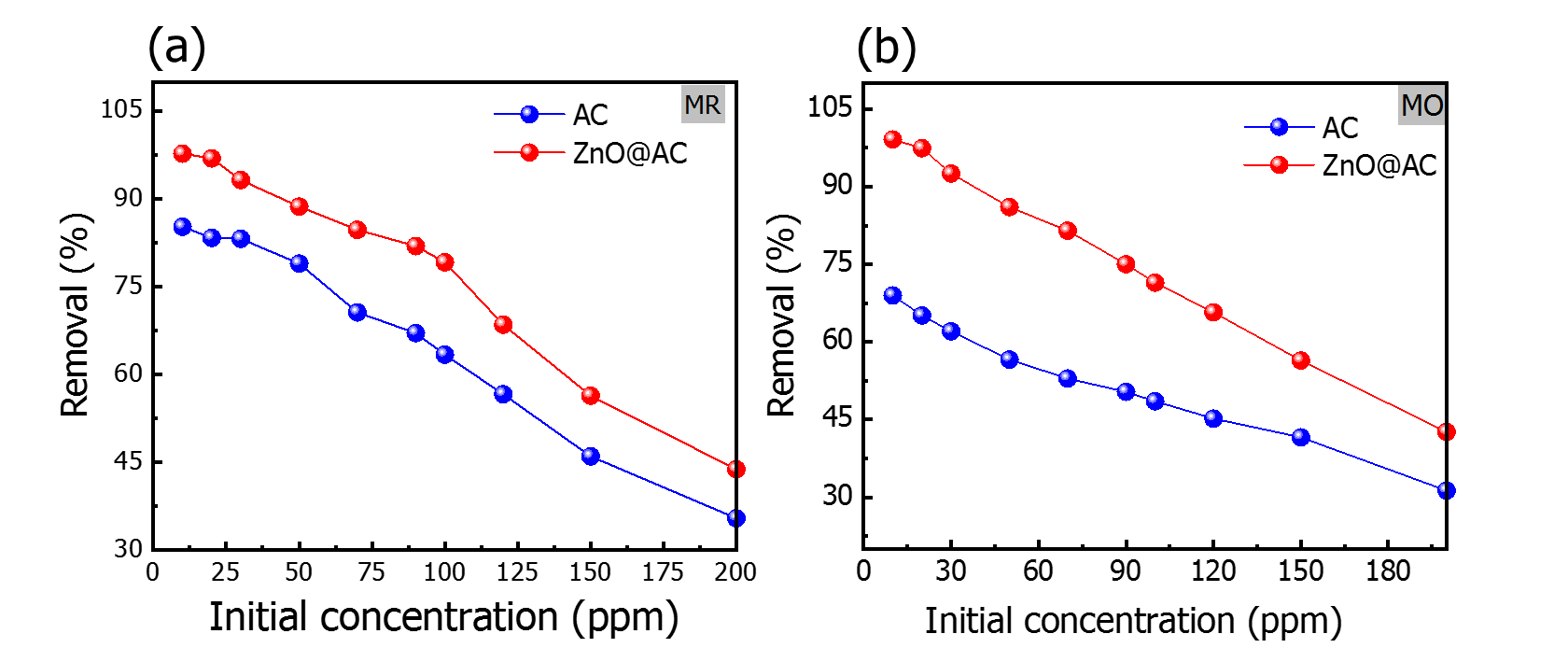


Figure S1: Effect of initial concentration of MR, and MO on the adsorption efficiency onto AC, and ZnO@ AC-derived sawdust
